# A successful endoscopic extraction of accidently ingested toothbrush in an adult: a case report

**DOI:** 10.1093/jscr/rjad044

**Published:** 2023-02-17

**Authors:** Ahmad Essam Al-Mulla, Fatemah Buhamad, Hawraa Fadel Marafie, Fatema Al Khalifa

**Affiliations:** Department of Surgery, Farwaniya Hospital, Ministry of Health Kuwait, Sabah Al-Nasser, Farwaniya, Kuwait; Department of Surgery, Farwaniya Hospital, Ministry of Health Kuwait, Sabah Al-Nasser, Farwaniya, Kuwait; Department of Surgery, Farwaniya Hospital, Ministry of Health Kuwait, Sabah Al-Nasser, Farwaniya, Kuwait; Department of Surgery, Farwaniya Hospital, Ministry of Health Kuwait, Sabah Al-Nasser, Farwaniya, Kuwait

**Keywords:** oesophagogastroscopy, toothbrush, flexible gastroscope, anorexia nervosa

## Abstract

Toothbrush ingestion is a rare phenomenon. It is usually found in psychiatric, elderly and mentally disabled patients. Foreign bodies usually pass spontaneously and uneventfully through the gastrointestinal tract. Nevertheless, larger objects may require early intervention to avoid complications. This report describes the course of treatment for a 25-year-old woman with an accidentally ingested toothbrush.

## INTRODUCTION

Foreign body ingestion is a typical emergency case [1]. Most small objects, such as coins, pins, marbles and buttons, pass through the stomach to the gastrointestinal tract without intervention. However, larger objects, such as toothbrushes that are 10 cm in length, cannot pass due to three narrow physiological spots: pylorus, duodenum C-loop and ileocecal valve [2]. These objects should be removed immediately to avoid pressure necrosis, intestinal obstruction or gastrointestinal perforation [3]. Here, we are presenting the case of a 25-year-old female with an accidentally ingested toothbrush.

## CASE REPORT

**Figure 1:**
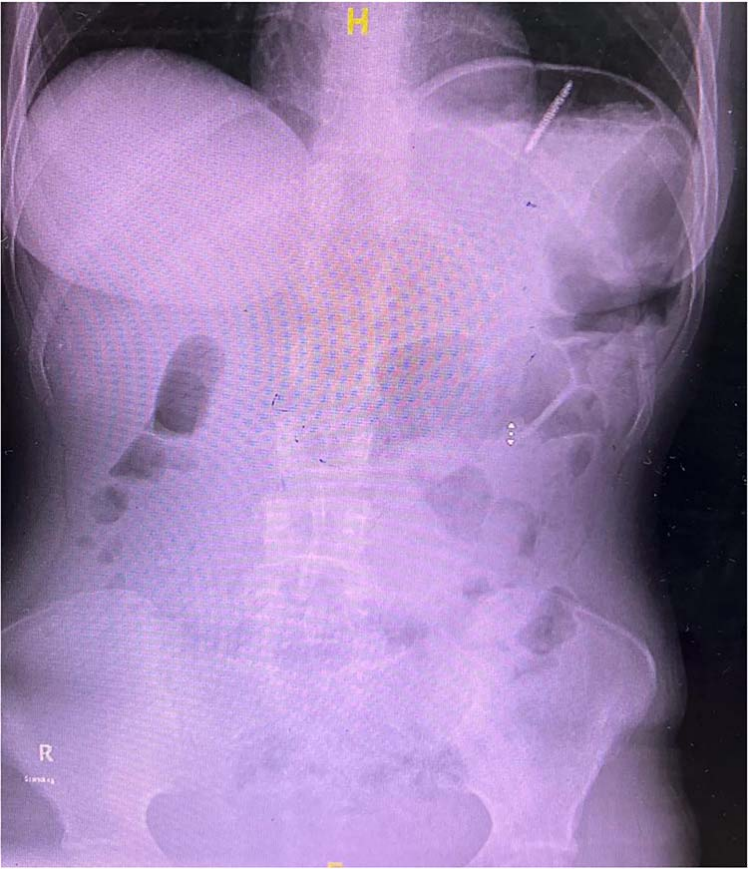
X-ray of abdomen showing the presence of a toothbrush at the left upper quadrant.

**Figure 2:**
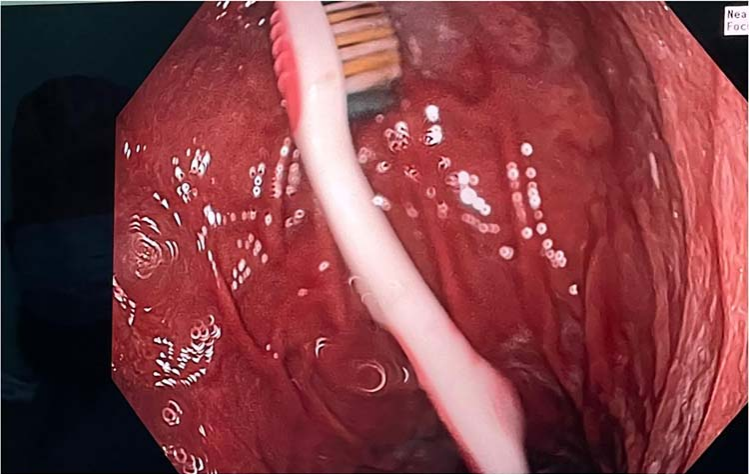
Toothbrush at the fundus of the stomach.

A 25-year-old female presented to the accident and emergency department with a history of accidentally ingested toothbrushes. The patient had a history of anorexia nervosa with multiple episodes of induced emesis. On presentation 3 hours post-ingestion, she was asymptomatic and vitally stable, with no physical abdominal findings. X-ray abdomen was ordered, which confirmed the presence of the toothbrush at the left upper quadrant (Fig. 1). The patient was counselled, and written consent was obtained for an upper oesophagogastroscopy extraction and possible surgery in case of failure of the initial procedure. The procedure was performed while the patient was under general anaesthesia, and the toothbrush was found at the fundus of the stomach (Fig. 2). It was extracted fully via a gastric balloon grasper by grasping the toothbrush from the head. A follow-up upper oesophagogastroscopy was conducted with no evidence of mucosal injury to the stomach or oesophagus. The procedure lasted for ~20 minutes. The patient was discharged after 6 hours and was referred to the psychiatry clinic for further assessment.

## DISCUSSION

Ingestion of a foreign body is commonly seen in children and adults with mental impairment, psychiatric illnesses and alcoholism as well as in elderly individuals who wear prosthetic dental materials [4]. However, toothbrush swallowing is rare. Approximately, 40 reported cases were mentioned in the literature only [5]. Foreign bodies in the stomach pass uneventfully in 80–90% of patients [6]. Nevertheless, objects 10 cm or longer, such as a toothbrush, may present difficulties due to the fixed retroperitoneal position of the C-loop of the duodenum [7]. In such cases, the foreign body should be removed as soon as possible to prevent complications such as bleeding, obstruction and perforation. The first reported death from ingested toothbrushes occurred in 1889 due to gastric perforation 3 days post-ingestion [8]. In another case report, the toothbrush penetrated the oropharynx of a young boy, and a broken part wedged close to the carotid artery [9]. Thirty-one case reports were reviewed with no spontaneous passage of toothbrush; all had to be extracted via an endoscope or a surgical procedure, depending on the experience of the gastroscopist. The first successfully reported procedure was in the 1980s [6, 10].

**Figure 3:**
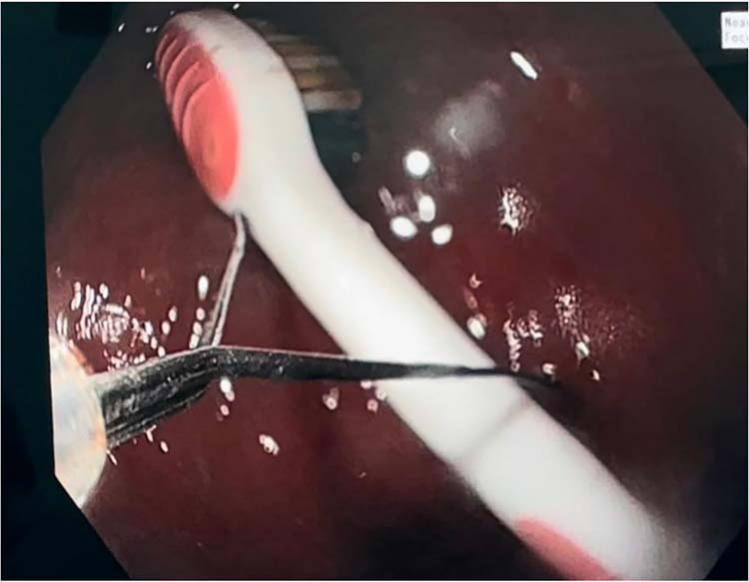
Toothbrush extraction via gastric balloon grasper.

In most cases, the preferred extraction tool was a basket or polypectomy snare. However, in our case report, we used a gastric balloon grasper to remove the toothbrush (Fig. 3). This had to be done by a skilled gastroscopist. It is wise to alter the position of the toothbrush such that it is in precise alignment with the oesophagus so that it can pass through the gastro-oesophageal junction. When the oropharynx is reached, it is wise to extend the head and use direct vision. In our case, manual manipulation was used to withdraw the toothbrush completely. In the possibility of extraction failure, it is preferred to be removed surgically, either laparoscopically or open surgery, depending on the surgeons’ experience and preference.

## CONCLUSION

Toothbrush extraction is a challenging case that requires an experienced and skilled gastroscopist. Early extraction is crucial to avoid morbidity and mortality. A failed endoscopic attempt may require laparoscopy or laparotomy to extract it successfully; thus, it is advisable to have a surgeon in the operating room.

## CONFLICT OF INTEREST STATEMENT

None declared.

## FUNDING

None.

## DATA AVAILABILITY

All data are available in the article.
